# Quantifying Media Effects, Its Content, and Role in Promoting Community Awareness of Chikungunya Epidemic in Bangladesh

**DOI:** 10.3390/epidemiologia2010008

**Published:** 2021-03-05

**Authors:** Mst. Marium Begum, Osman Ulvi, Ajlina Karamehic-Muratovic, Mallory R. Walsh, Hasan Tarek, Jailos Lubinda, Alfonso J. Rodriguez-Morales, Shehzad Sarfraz, Jesús A. Treviño C, Muhammad Yousaf Shad, Ubydul Haque

**Affiliations:** 1 Department of Pharmacy, East West University, Dhaka 1212, Bangladesh; mariumpharm23@gmail.com; 2 Department of Public Health and Prevention Sciences, Baldwin Wallace University, Berea, OH 44017, USA; mwalsh17@bw.edu; 3 Department of Sociology and Anthropology, St. Louis University, St. Louis, MO 63108, USA; ajlina.karamehicmuratovic@slu.edu; 4 Department of Pharmacy, Dhaka International University, Dhaka 1213, Bangladesh; hasantarek93@gmail.com; 5 School of Geography and Environmental Sciences, Ulster University, Coleraine BT52 1SA, UK; jailoslubinda@gmail.com; 6 Grupo de Investigacion Biomedicina, Faculty of Medicine, Fundacion Universitaria Autonoma de las Americas, Pereira, Risaralda 660003, Colombia; alfonso.rodriguez@uam.edu.co; 7 Department of Current Affairs and Programming, DAWN News TV, Islamabad 44000, Pakistan; shehzadnumlian@gmail.com; 8 School of Architecture, Universidad Autónoma de Nuevo León, San Nicolás de los Garza, NL 66455, Mexico; jtrevino41@hotmail.com; 9 Department of Statistics, Quaid-i-Azam University, Islamabad 44000, Pakistan; yousuf@qau.edu.pk; 10 Department of Biostatistics and Epidemiology, University of North Texas Health Science Center, Fort Worth, TX 76107, USA; mdubydul.haque@unthsc.edu

**Keywords:** Alphavirus, Facebook, epidemic, social media, Bangladesh

## Abstract

Background: Chikungunya is a vector-borne disease, mostly present in tropical and subtropical regions. The virus is spread by *Ae. aegypti* and *Ae. albopictus* mosquitos and symptoms include high fever to severe joint pain. Dhaka, Bangladesh, suffered an outbreak of chikungunya in 2017 lasting from April to September. With the goal of reducing cases, social media was at the forefront during this outbreak and educated the public about symptoms, prevention, and control of the virus. Popular web-based sources such as the top dailies in Bangladesh, local news outlets, and Facebook spread awareness of the outbreak. Objective: This study sought to investigate the role of social and mainstream media during the chikungunya epidemic. The study objective was to determine if social media can improve awareness of and practice associated with reducing cases of chikungunya. Methods: We collected chikungunya-related information circulated from the top nine television channels in Dhaka, Bangladesh, airing from 1st April–20th August 2017. All the news published in the top six dailies in Bangladesh were also compiled. The 50 most viewed chikungunya-related Bengali videos were manually coded and analyzed. Other social media outlets, such as Facebook, were also analyzed to determine the number of chikungunya-related posts and responses to these posts. Results: Our study showed that media outlets were associated with reducing cases of chikungunya, indicating that media has the potential to impact future outbreaks of these alpha viruses. Each media outlet (e.g., web, television) had an impact on the human response to an individual’s healthcare during this outbreak. Conclusions: To prevent future outbreaks of chikungunya, media outlets and social media can be used to educate the public regarding prevention strategies such as encouraging safe travel, removing stagnant water sources, and assisting with tracking cases globally to determine where future outbreaks may occur.

## 1. Introduction

Chikungunya infection is an alarming health hazard, occurring mostly in tropical and subtropical regions, with over one billion people living under its risk [1]. It is a viral, vector-borne disease, mainly transmitted by *Ae. aegypti* and *Ae. albopictus* mosquitos from the Togaviridae family of viruses [2]. While chikungunya was originally only a risk to people in parts of Africa [3] with the first diagnosis in Tanzania in 1952 and transmitted by *Ae. aegypti* mosquitos, it has evolved to give *Ae. albopictus* mosquitos the capability to spread it. Most people infected with the virus develop symptoms 2–7 days after a bite, and common symptoms include high fever, rash, debilitating joint pain, gastrointestinal issues, and headaches [2]. Most of the post-acute disease includes severe pain in hands, wrists, knees, ankles, and feet that can last for years [4]. Patients also report lingering symptoms such as fever, rash, headaches, fatigue, and gastrointestinal manifestations [4]. The extreme joint pain can be chronic and could potentially lead to long-term health effects such as arthritis. Arthralgia has also been reported three months’ post-infection [4]. The cumulative incidence of post-acute disease of chikungunya six months’ post-infection was 42.9% [5]. However, other studies have reported up to 59% after years [6,7,8,9].

One region impacted by this virus, Dhaka, the capital of Bangladesh, is hot and humid, providing an ideal climate for breeding mosquitos. Dhaka is also home to more than 18 million residents and it experienced an epidemic of chikungunya infection in 2017 [10,11]. This epidemic lasted from April to September and it has been hypothesized that actual chikungunya cases were much higher than reported cases [10]. The effects of the chikungunya outbreak in 2017 left devastating effects on both the economy and quality of life [12].

The Ministry of Health in Bangladesh established a 24/7 hotline to better assist with the management of this epidemic and to create mass awareness to prevent the disease. The mainstream and social media played an important role in building public awareness, diagnosis, treatment, and prevention of chikungunya. Effective prevention strategies include learning more about the environmental conditions suitable for *Ae. aegypti* and *Ae. Albopictus* mosquitos, as well as learning how to keep these mosquitos away. In this study, we investigated the role of mainstream and social media in the prevention and control of the epidemic.

## 2. Methods

We collected chikungunya-related information provided from the (1) top nine television channels airing from 1st April to 20th August 2017, (2) all the news published in the top six dailies in Bangladesh, (3) the 50 most viewed chikungunya-related Bengali videos (manually coded and analyzed for analysis) and (4) other social media outlets, such as Facebook. For the average (arithmetic mean), we computed the ratio of total social community responses with cumulative number of days (time) for all such posts (average = (sum of all comments, likes etc.)/(no. of days of post1 + no. of days of post2 + …….)) e.g., if we have 1000 comments on 10 posts which have been posted on 10th June, 9, 8, 7,…1st June, the average comments per post per day will be (1000/(10 + 9 + 8 + ….. + 1)).

## 3. Statistical Analysis

We plotted the data curves of chikungunya by the number of weekly cases reported from the 1st week of April to the last week of September 2017. The activities on national media in the form of television news, talk shows, promos, public awareness posts, and advertisements were followed, and community response considering the above four sources were graphed by weeks to estimate how the public reaction is changing to reciprocate the chikungunya epidemic situation over time. We used a generalized linear model (GLM) (the covariates are news, shares, likes etc.) approach to analyze the data [13]. The GLM regression model allows relating the response variable (for example cases) via a link function (for example log), and also the size of the variances of each exploratory into the predicted value of study variable (understandable by users of regression models).

Each observed value of the study variable **Y** (dependent variables such as cases) is assumed to follow a particular exponential (social media posts are independent behavior of community). When you post something, it receives a response which reduces over time following an exponential model, it is understandable by all data scientists, distribution of behavior over time always follows an exponential model, e.g., waiting time distribution that includes the normal, binomial, Poisson and gamma distributions. The GLM consists of the following three elements:

1. A probability distribution from the exponential family (the normal, binomial, Poisson and gamma distributions etc.).
2. A linear predictor **X*β***, where **X** is an ‘***nxp***’ data matrix of ***p*** independent variables and ***β*** is a vector of unknown parameters which is estimated from data.
3. A link function *g* such that

E(**Y**) = *g*^−1^(**X*β***). (1)

The exponential family of distributions is commonly used for practical modeling of discrete (such as count data) and continuous data. The exponential family of distributions is a natural choice for many mathematical phenomena. The set of predictors X incorporates the information of explanatory variables through their coefficients vector ***β***, which is related to the value of the study variable (Y) (chikungunya cases) through a link function. The link function provides the relationship between the linear predictor and the mean of the distribution function (E(Y)). The choice of link function from many commonly available link functions depends upon how well it is defined and how it relates to the exponential of the response’s probability mass function [13].

As soon as some news about chikungunya cases aired, social media self-activated to disseminate awareness to the public. We investigated if there is any relation between our study variable (chikungunya cases) and the effectiveness of social media campaigns to reduce the chikungunya cases. The necessary covariates by expanding the above data by weeks. This methodology focused on weekly data (Y is the study variable, i.e., chikungunya cases. Social media posts are independent behavior of community). When you post something, it receives a response that reduces over time (follows an exponential model, it is understandable by all data scientists, distribution of behavior over time is always follow exponential model e.g., waiting time distance, we added reference as well). The covariates are news, shares, likes etc.:

Surveillance of certain outbreaks follows an exponential pattern of responses [14] over time. Following equation 1, it computes the cumulative chance of occurring a response over the lifetime *x* with some rate of occurrence µ [15].

F(*x*,µ) = 1-e^−*µx      *^
*for x ≥* 0 and zero otherwise. (2)

Whenever a surge occurs, responses start quickly, which reduces over time until some new surge (cases) occurs. This gives space to emerging a recency error. An event (surge) draws its attention immediately, continuing for a period of time and then getting occupied by new events in the media. Considering this factor, our estimates use the occurrence of cases for a few successive periods cumulatively moving the past events.

The model with a particular set of covariates further investigates which one is significant. The last column in Table 2 (*p*-values) shows this relationship. This column represents the results that among the set covariates, the probability (*p*-value) that an estimate may get the higher value (of the test statistic) than is estimated from the given set of data. There is no direct comparison among the contributing covariates. It only sheds light on the strength of significance of a particular covariate when the given set of a covariate is selected in the model fit (the Akaike information criterion (AIC)). In a different model fit with a different set of covariates, the same covariate may yield a different *p*-value. A smaller *p*-value is considered as the more significant covariate to estimate the study (fitted) variable.

## 4. Results

**Figure 1:**
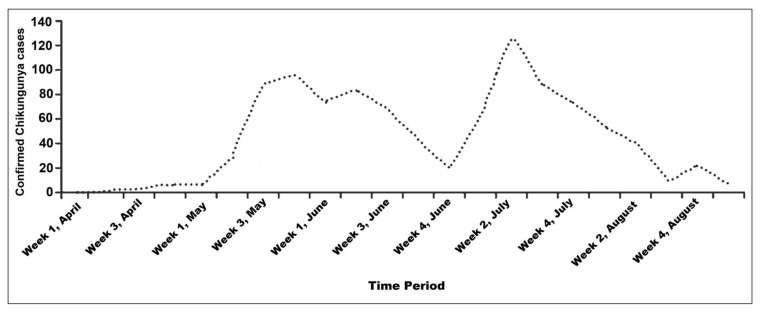
Chikungunya epidemic (cases confirmed by polymerase chain reaction (PCR)) in 17 districts of Bangladesh, 1 April–7 September, 2017.

During the chikungunya outbreak in 2017, two large peaks of the outbreak were observed. Starting from the first week of April, chikungunya infection remained dormant until mid-May when the epidemic soared to nearly 100 confirmed cases [10]. The second and highest peak of over 120 confirmed cases was observed during the second week of July [10]. The epidemic steadily decreased by the fourth week of August, and by the first week of September, confirmed cases of chikungunya were null [10] (Figure 1).

A total of 269 news articles on chikungunya were published in the top six printed dailies in Bangladesh. Among these, 53% were reports, 20% awareness posts, 21% advertisements, and the rest were editorials discussing chikungunya outbreak control (Table 1). These news articles focused on the control of chikungunya and the epidemiological aspects of the disease. Furthermore, six daily newspapers contributed to the public awareness campaign with reports, news, awareness posts, advertisements, and editorials (Table 1). There were also 320 talk shows, promos, public awareness posts, and advertisements from the mainstream media (Table 1).

There were likes/reactions (3.23 million), shares (0.74 million), and comments (0.004 million) on posts or blogs regarding chikungunya (Table 1). These reports and posts were published in four popular online Bengali portals during the outbreak. A total of 153 news reports regarding the chikungunya onslaught were published online.

Popular sources such as the top six dailies in Bangladesh, local news outlets, and Facebook, spread awareness of the outbreak on the internet with the goal of reducing cases of chikungunya. Facebook and other social media outlets revealed the number of chikungunya-related posts and responses to these posts (Table 1).

**Figure 2:**
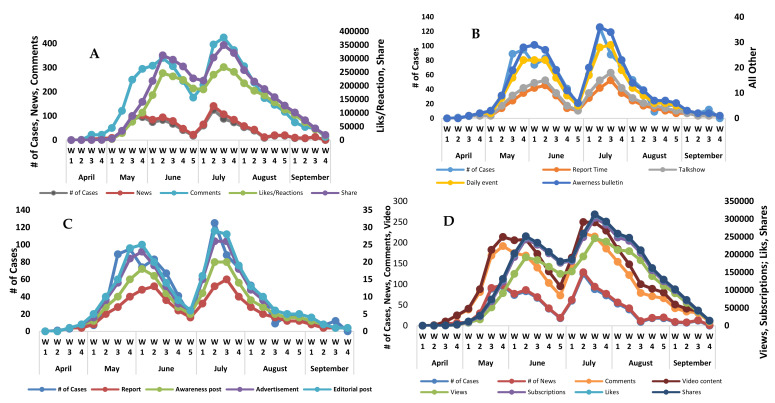
Various data series plot with actual cases reported ((**A**). Web resources, (**B**). TV Channels, (**C**). Daily News Papers, (**D**). YouTube Resources).

The media play a major role and have a huge impact on the way that news events are defined and perceived. An event, such as an outbreak of chikungunya, means media reports, videos, and awareness promos based on cases are reported. The response of the social media community to these media posts follows an exponential distribution [14] and typically decreases over time, as was the case with chikungunya in 2017 (Figure 2). The rate of occurrence is reciprocal on average, which can depend on multiple of all such live posts (a post may live for an infinite time, generally as long as the issue alive). Successive posts on some live issues show similar behavior of followers and collective social community response accumulated as the sum of exponential series which follow an exponential behavior [15].

AIC of a log-likelihood model is penalized for a number of covariates. Its lower value suggests a “better” model, but it is a relative measure of model fitness. On the basis of AIC, the electronic and print (EP) media model is the best fit (Table 2) following the news channels model. Internet resources model is the worst fit among the models given in Table 2 with AIC 527. These results are presented in the data over 26 weeks (outbreak period). A large dataset may compute better results. The AIC allows comparisons of different models estimated on the same dataset (26 weeks) with various choices of the set of covariates (e.g., news, shares, likes etc.).

From the Table 2, if we consider the model “Electronic and Print Media”, news TV awareness bulletins, newspapers awareness posts, and newspaper advertisements are equally significant (*p*-value = 0.0000) at estimating the number of cases when we use all eight covariates. In the same model, newspaper editorials are also significant but not as much as the above three. In the first model (News Channels, related four covariates each), daily events and reports are significant with *p*-value 0.0000 and 0.0014, respectively, whereas, with the “Daily newspapers” model (related four covariates each), reports and advertisements are significant covariates. These significant covariates are different from their collective “Electronic and Print Media” model. Only the newspaper advertisement is significant in the “Daily newspapers” model and “Electronic and Print Media” model. This behavior (significance or non-significance) of a covariate(s) in a small or large set of variables, the model due to interdependence of variables cannot decide which of the collinear covariate actually may be responsible for the changes in the fitted variable. Moreover, a negative confounder effect may come into play which artificially reduces the association between a study and covariate variables.

Figure 2 shows various models, i.e., web resources of four websites, activities on YouTube, TV news channels broadcasting activities, daily newspaper campaigns, news coverage on internet resources (YouTube and websites), and electronic and print (TV channels and newspapers) media coverage. As soon as the reports populated the print, electronic, and social media, civil society started their response on various outlets. Different proportions of masses used various news resources and some people used multiple information resources at the same time. The electronic and print (EP) media line is closest to actual cases.

## 5. Discussion

Media has historically played an important role in conveying important information about health to the public. As soon as the reports of chikungunya received media attention, social media started discussing, sharing reports, videos, and comments on various posts, as well as re-posting (Table 2). As the epidemic got worse, these activities also increased, including at an individual level with people sharing their experiences with neighbors, colleagues, relatives, friends, and most importantly on social media sites. The social amplification of risk framework (SARF) is a phenomenon where information processes, institutional structures, social behaviors, and individual responses shape the social experience of risk [16]. The SARF is a useful framework through which to consider the chikungunya epidemic in Dhaka, as it helps explain the role that social media played in amplifying public attention to spread awareness of the outbreak, eventually reducing cases of chikungunya, and showing that media outlets have the potential to impact future outbreaks of these alphaviruses.

This research work set its goal to analyze how media play their role while in an epidemic spread. When an epidemic starts, there is poor awareness among the masses. The spread enlarges its scale which produces worries on social media and mass media. People’s awareness increases, they adapt control measures, which reduces the scale of reported cases. This is obvious from data (see pi 1 and 2) where we plot reported cases, number of news articles, comments, shares, videos, vies, subscriptions and likes, etc. over time horizon of epidemics. Our hypotheses were how media play their role to control the disease by creating awareness among masses. We achieved this goal by fitting an appropriate probabilistic model which can be used to predict the scale of the epidemic spread over time. These fitted models on various media aspects are very much consistent with actual disease data. The AICs provide a strong evidence of their goodness of fit (see Table 2). We are providing sufficient evidence that our fitted models are good, and they help us to support and elaborate our study goals and suggested hypotheses. Most of these models are significant (see *p*-values, Table 2), which leads us to good, confident, and comprehensive model selection while predicting the epidemic.

Most of the population covered by this media used social media as a source of information. Media surveillance of certain outbreaks follows an exponential pattern of responses [13] over time. This is initially a high response which reduces over time.

This electronic print (EP) media section has access to almost all people belonging to every walk of life, various ethnic groups in villages and cities, poor and rich people. The proportion of accessible masses may not be as much as EP media. However, model fit results with other models are as good as the EP model. A model with a low AIC does not mean it is a correct model, but it is better compared to others due to poor quality and overfits. The idea behind AIC is a model with a lesser number of parameters is good and consistent. Our model reduced the error degree of freedoms, i.e., the independent observations to compute AIC. All these sets of covariates show similar line drawings as all these predictors (independent variables, e.g., news, shares, likes etc.) show a similar rise or fall directly in the data.

Much of the affected population encountered negative experiences during this epidemic, as there was only one institution, the Institute of Epidemiology, Disease Control and Research (IEDCR), that was equipped with the diagnostic facilities for the chikungunya infection [10].

From previous studies it has been found that stagnant water, unseasonal heavy rainfall due to climate change, unplanned and poor urbanization, frequent trade, and travel and clogged sewerage systems are some key concerns of the chikungunya infection [2,17,18]. The media should disseminate information on preventive strategies for any future epidemics of chikungunya by airing more educational advertisements, promos, talk-shows, campaigns, etc. Print, electronic and social media should emphasize removing any water-holding containers in peoples’ yards or houses [19]. Travel is another factor that may encourage the spread of these mosquitos, especially through air travel, introducing the virus to new regions [20]. Therefore, the media can encourage participation in pre-travel counseling on the prevention of chikungunya [21]. Improving communication and education regarding safe travel is key to controlling the spread of chikungunya.

Chronic cases of chikungunya may have symptoms that last months or years such as arthritis [7]. Prevention techniques such as encouraging pre-travel counseling for safe travel and removing objects holding stagnant water are keys to controlling outbreaks of chikungunya and other alphaviruses. Using media outlets more frequently with adequate prevention methods may prevent future epidemics.

**Figure 3:**
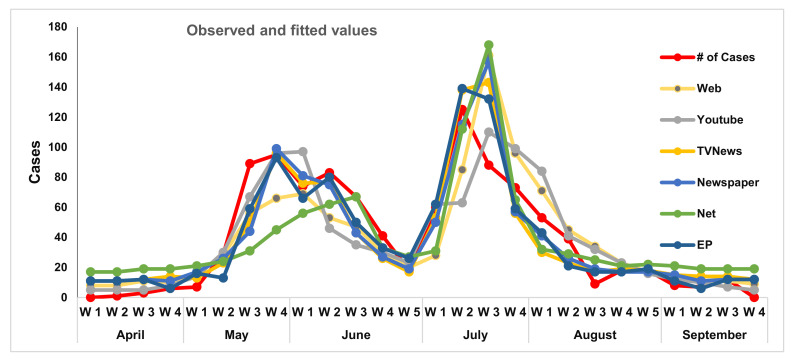
Observed cases are shown with fitted values of various models fit.

This study indicates that an increase in social media overlays the decreasing number of cases of chikungunya. Figure 3 represents observed cases along with fitted cases by fitting models using web resources1 exploratory variables (web news, Likes/Reactions, Shares and Comments), YouTube2 exploratory variables (Likes, Comments, Shares and Video content lengths (Minutes)), TV-News3 exploratory variables (Video content lengths (Minutes), Report Time (5–10 min), Talk shows (1–1.5h), Daily events (1–3 min) and Awareness bulletins (45–120 s)), newspapers4 exploratory variables (Report, Awareness post, Advertisements), internet TV5 exploratory variables (no of News, Views of Channels and Subscriptions) and Editorial Posts6 (Editorials and reports).

Beginning the second week of April, reports about the epidemic spread and started to appear into news. Reports reflected the rise in the number of cases. The population became aware and started following the disease reports on various media outlets. At the end of June (after completing the first cycle), the frequency of reports and other activities reduced, however, the threat was not completely eradicated.

The second phase of the disease started with more vigor. It went on rising and reached its peak during the third week of July. Additionally, people’s reactions increased and awareness/mitigation strategies formulated.

From the third week of July, when the entire nation was made aware, the reports of cases reduced, and it was completely over by mid-August. After that, there were reports on the usual number of cases without a threat of the spread of disease.

It is important to mention that quantifying the actual time social media users spend reading is challenging. There are metrics measuring this, however, it is beyond the scope of our manuscript. Even if a reader interacted with the article through likes, shares and comments etc., it does not necessarily mean they have read and understood the material.

In March of 2020, the WHO declared the COVID-19 outbreak a global pandemic [22]. Global lockdowns required citizens to start spending more time at home, and as a result, media consumption has both increased and changed [23]. The COVID-19 pandemic serves as another good example of the role that social media can play in health outcomes. For instance, a recent quantitative survey study conducted by Krawczyk et al. (2020) offered data and analysis of online news media coverage of COVID-19. Twenty-six million news articles from the front pages of 172 major online news sources in 11 countries were collected [24]. The authors found that COVID-19 coverage accounted for approximately 25% of all front-page online news articles between January and October 2020 [24]. Further results suggest that an information overload in COVID-19 reporting could risk obscuring effective policy communication [24].

Cinelli et al. (2020) addressed the diffusion of information about the COVID-19 with a massive data analysis on Twitter, Instagram, YouTube, Reddit and Gab. Engagement and interest in the COVID-19 topic and a differential assessment on the evolution of the discourse on for each platform and their users were analyzed [25]. The authors found that information from both reliable and questionable sources does not present different spreading patterns [25].

It is likely that going forward, quantifying in media the effects of the COVID-19 pandemic will be studied well into the future.

## 6. Conclusions

Chikungunya has had a significant impact on human health and continues to be a public health concern. The mainstream and social media outlets have been associated in reducing the fatality of chikungunya by delivering awareness messages to the public and tracking where the virus spreads. Media outlets were active with managing the control of the chikungunya outbreak in Bangladesh in 2017. It is imperative that media outlets continue to relay information to the public to avoid a future outbreak of chikungunya or other alphaviruses, as well as to improve public health infrastructure utilizing these technological advances to prevent outbreaks in Bangladesh and elsewhere. Major outbreaks have occurred in the past decades that have brought the incidence of chikungunya to approximately one million cases per year globally. These cases may have been avoided or minimized had media outlets been utilized more frequently or actively prior to or during these outbreaks. In the future, we must put a significant emphasis on encouraging media outlets and social media to utilize their platform and educate people regarding prevention strategies such as safe travel and removing sources of stagnant water both indoors and outdoors, as well as using media to track regions where outbreaks of chikungunya and other alphaviruses are common.

## Figures and Tables

**Table 1:** Chikungunya related information from various resources.

Websites Sources						
Website	Total news	Reactions		Share	Comments	
Bangla tribunes	61	1,678,980		345,000	2400	
Jagonews24.com	31	498,022		150,402	346	
Bdnews24.com	22	598,340		157,600	590	
Poriborton.com	21	449,821		90,765	221	
TV Channels						
Channel	Report (5 to 10 min)	Talk show (1 to 1.5 hr)		Daily events	Awareness post	
Chanel I	15	3		0	14	
ATN Bangla	17	3		25	5	
nTV	6	1		0	7	
Independent	6	2		45	2	
Ekattor	20	5		0	17	
Banglavision	10	3		0	2	
News 24	50	3		0	3	
Somoy News	10	3		35	0	
BBC	2	3		0	3	
Daily newspaper						
Daily	Reports	Awareness post		Advertisement	Editorial post	
Prothom alo	36	19		4	2	
Ittefaq	18	16		18	8	
Bangl. Protidin	28	9		25	5	
The Daily star	13	4		5	2	
The Observer	26	4		1	1	
The Independent	22	1		1	2	
Contributing news sources in YouTube						
Source in YouTube	news	Views	Subscript.	Like	Comnts.	Shares
TV channels based	24	1,560,003	259,009	76,521	590	7004
You Tube TVs	20	853,247	234,460	24,690	333	5583
Health professional video contents	6	487,960	99,305	4,865	557	6523

**Table 2:** Akaike information criterion (AIC), Covariates and *p*-values in various models.

Model	AIC	Covariates	*p*-Value
Web Resources	366.73	News	0.0017 **
		Likes	0.0001 ***
		Shares	0.0074 **
		Comments	0.0000 ***
YouTube Resources	328.91	News	0.0002 ***
		Likes	0.5940
		Shares	0.7392
		Comments	0.0000 ***
		Views	0.0001 ***
News Channels	323.47	Reports	0.0014 **
		Talkshow	0.2701
		Daily events	0.0000 ***
		Aware bulletins	0.2274
News Papers	328.88	Reports	0.0000 ***
		Awareness posts	0.0294 *
		Advertisements	0.0003 ***
		Editorials	0.0992
Internet Resources	526.99	Web news	0.0000 ***
		YouTube news	0.3700
Electronic and Print Media	294.39	TV reports	0.1304
		TV talkshow	0.0890
		TV daily events	0.5509
		TV aware bulletins	0.0000 ***
		P. reports	0.0628
		P. awareness posts	0.0000 ***
		P. advertisements	0.0000 ***
		P. editorials	0.0084**

Significance (strength) codes: 0, ‘***’, 0.001 ‘**’, 0.01 ‘*’, 0.05 ‘^.^’, 0.1 ‘ ’ and 1.

## Data Availability

Data supporting the conclusions of this manuscript are provided within the article and will be available from the corresponding author upon request.

## List of Abbreviations

GLM = generalized linear model; Y = Dependable variable; EP = electronic and print; SARF = Social amplification of risk framework; IEDCR = Institute of Epidemiology, Disease Control and Research; PCR = Polymerase chain reaction; AIC = Akaike information criterion.
